# Recent Advances in Functional Polymer Materials for Water Treatment

**DOI:** 10.3390/polym17121654

**Published:** 2025-06-14

**Authors:** Zhiwei Wang, Tongtong Wang, Yanzhu Guo, Jian Zhang

**Affiliations:** 1 Key Laboratory of Clean Pulp & Papermaking and Pollution Control of Guangxi, College of Light Industrial and Food Engineering, Guangxi University, Nanning 530004, China; zhangjian@gxu.edu.cn; 2 Institute for Interdisciplinary and Innovation Research, Xi’an University of Architecture and Technology, Xi’an 710055, China; 3 The Liaoning Province Key Laboratory of Paper and Pulp Engineering, College of Light Industry and Chemical Engineering, Dalian Polytechnic University, Dalian 116034, China; guoyz@dlpu.edu.cn

## 1. Introduction

With the increasing severity of global water pollution, research on the application of functional polymer materials in the field of water treatment is receiving increasing attention [1]. Due to their unique physicochemical properties, such as their large molecular weight, controllable structure, large specific surface area, and easy modification, functional polymer materials have shown great potential in water treatment. These materials can not only effectively remove pollutants in water, such as heavy metal ions, organic dyes, and microorganisms, but also enhance their separation efficiency and stability through modification [2,3,4]. Recently, with the rapid development of materials science, new flocculants, scale inhibitors, functional filtration membranes, ion-exchange resins, selective adsorption materials, modified functional fibers, and other polymer materials have been emerging [2]. These materials allow for the efficient removal of pollutants and the recycling of resources. Moreover, the application of nanocomposites and hybrid nanomaterials in microbial fuel cell electrodes has received increasing attention, opening up new research directions in the field of water treatment [4,5,6]. Functional polymer materials have been widely used in the field of wastewater pollution control, and their diversity and tunability provide new ideas and methods for solving the increasingly serious water environment problems [3]. The research on such materials not only promotes the innovation of pollutant treatment technology, but also promotes the practice of green chemistry and the concept of sustainable development in the field of water treatment.

Moreover, research on the application of functional polymer materials in water treatment has made significant progress. Regarding membrane materials, researchers are committed to developing new polymer membranes with higher selectivity, flux, and stability, and exploring their applications in seawater desalination, industrial wastewater treatment, and other fields [3,4]. For example, porous core–shell nanofiber membranes exhibit highly efficient photocatalytic performance, while new functional membranes based on graphene oxide achieve excellent dye retention through layer spacing modulation [5]. As for adsorbent materials, the synthesis and modification of novel polymeric adsorbents are hot research topics, such as the efficient adsorption of heavy metal ions by ionic liquid cross-linked hydrogels and the green synthesis method of magnetic chitosan composites, which provide innovative solutions for the treatment of heavy metals and organic pollutants [4]. Furthermore, research on polymer-based photocatalytic materials and biodegradable polymer materials has also gained extensive attention in recent years, which has culminated in the efficient removal of pollutants in water through photocatalytic degradation, selective adsorption, and bio-enhanced action [6,7]. These research advances promote the innovation of water treatment technology and lay a solid foundation for developing more efficient and environmentally friendly solutions.

This Special Issue aims to collect and publish high quality research results on the latest advances in functional polymer materials for water treatment. We encouraged the submission of original articles, review articles, case studies, and newsletters on polymeric flocculants and adsorbents, polymeric separation membranes and ion exchange resins, polymeric materials for enhanced biological water treatment, polymeric materials for water treatment equipment, and the preparation and modification of polymeric materials. Through this Special Issue, we expect to provide the latest research results on functional polymer materials in water treatment and further promote the development of this field.

## 2. Overview of the Published Articles

A total of 19 submissions were received, and 10 articles were accepted for this Special Issue. These articles cover various aspects of membrane materials, adsorbent materials, photocatalytic materials, and biological treatment materials. From the optimized design of membrane materials to the preparation and performance study of adsorbents, and then to the exploration of the application of photocatalytic composites and biofilm carriers, these articles provide an in-depth discussion of the synthesis, modification, performance characterization, and practical application of the materials. This Special Issue focuses on the application of functional polymeric materials in the field of wastewater pollution control, discusses the characterization of the physicochemical properties of functional polymeric materials based on various advanced techniques, and provides insights into their removal effects and mechanisms. The articles in this Special Issue not only demonstrate the potential application of functional polymer materials in the treatment of polluted wastewater, but also provide researchers in the related fields with a theoretical basis and practical experience with important reference value. It is expected that this Special Issue will promote the further development of functional polymer materials in the field of water treatment and contribute to solutions to the global water shortage and water pollution problems.

This Special Issue systematically demonstrates the frontier breakthroughs of functional polymeric materials in the field of water treatment. In heavy metal treatment, Sun et al. (contribution 2) developed an ionic liquid cross-linked hydrogel (PAM/AA/[Vim]Br_2_) that removed 98.1% of Cr^3+^ at 100 ppm via physico-chemical dual mechanism adsorption, and its XPS analysis revealed that the coordination of carboxylate groups with amino groups dominated the adsorption at high concentrations. Fakhry et al. (contribution 3) further extended the application of bio-based materials and found that the maximum adsorption capacity of Cr(VI) by fungal melanin (*Aureobasidium pullulans*) reached 595.974 mg/g, which was a 22.7% enhancement compared to the conventional biomass, and the prediction error of the adsorption process was reduced by 37% through decision tree modeling. Yadav et al. (contribution 4) focused on green synthesis techniques and prepared magnetic chitosan composites (MCSs) using *Myrica esculenta* leaf extracts with Cd^2+^ adsorption up to 426 mg/g, retaining 80.44% efficiency after seven cycles and a 69.5% increase in the BET specific surface area (178 m^2^/g) compared to unmodified magnetic nanoparticles. In the field of membrane separation technology, Yang et al. (contribution 6) constructed AgCl/ZnO heterogeneous nodule shell nanofiber membranes via coaxial electro spinning, which achieved a 98% photodegradation of methylene blue in 70 min, whilst the efficiency still exceeded 95% after five cycles. Moreover, Meng et al. (contribution 9) modulated graphene oxide (GO) membrane layer spacing via ethylenediamine (EDA) cross-linking, which enhanced the dye retention to 96% and increased the permeate flux by 5-fold, whilst XPS confirmed that C-N covalent bonding inhibited membrane swelling. For biological treatment enhancement, Liu et al. (contribution 10) used a surface positively charged modified polyurethane carrier (+1.79 mV) to increase the abundance of anaerobic ammonia-oxidizing bacteria (AnAOB) by 2.3-fold, which resulted in a 78% removal of the total nitrogen in an SBR reactor, and reduced the nitrate concentration to 6.03 mg-N/L. These results confirm the theory of the “multi-mechanism synergistic removal of pollutants” and provide a new paradigm for the management of complex pollutants.

In the fields of engineering applications and smart material design, the articles in this Special Issue show remarkable innovations. Wei et al. (contribution 5) developed a cationic polyacrylamide (CPAM) suspension for oilfield wastewater treatment problems, which reduced the dissolution time by 81.4% (16 min) and achieved a 35.6% turbidity removal rate via a continuous grafting process with a dual initiator, which was 25.4% higher than the traditional solid CPAM. Xie et al. (contribution 7) focused on the decolorization of indigo wastewater. The PP-g-AA-MAH fiber prepared with a dual initiator (DBPH/BPO) had an adsorption capacity of 110.43 mg/g for indigo, which maintained 81.7% efficiency after eight cycles, and FTIR confirmed the successful grafting of acrylic acid with maleic anhydride (contribution 7). For scale inhibitor materials, Al-Hamzah et al. (contribution 8) systematically evaluated sulfonated polycarboxylic acid scale inhibitors (molar mass 2000–9500 g/mol). They found that 2000–2500 g/mol of low-molecular-weight materials inhibited calcium sulfate deposition with 85% efficiency in seawater at 125 °C, which provides key data for the optimization of the thermal desalination process. Smart responsive material design is a highlight: a review by Kumar et al. (contribution 1) shows that MOFs/COFs hybrid systems increase the membrane selective permeability to 3–5 times that of conventional polymers. Innovations in characterization techniques are also prominent, with Fakhry et al. (contribution 3) introducing decision tree modeling for the first time to optimize the Cr(VI) adsorption process. Additionally, Meng et al. (contribution 9) analyzed the amidation mechanism of EDA with GO via in situ FTIR-XPS coupling.

These results not only break through the theoretical framework of dynamic adaptive materials, but also promote the intelligent water treatment system on the ground. Future research can focus on material database construction and machine learning linkage, such as the neural network optimization of adsorption strategies (contribution 3) and full life cycle assessment (LCA) system, which together promote the development of water treatment technology towards precision and sustainability (contribution 2).

## 3. Conclusions and Outlook

This Special Issue focuses on the latest progress in functional polymer materials in the field of water treatment, and through the inclusion of 10 high-quality articles, it comprehensively demonstrates the remarkable effectiveness and innovative applications of functional polymer materials in solving water pollution problems. These papers not only cover research on polymer membranes, adsorbent materials, anti-scaling agents, and other aspects, but they also discuss the preparation technology, performance optimization, and practical application effects of the materials. By synthesizing the conclusions of the papers, it is clear that functional polymer materials, by virtue of their unique physicochemical properties, have demonstrated excellent performance in many aspects, such as heavy metal ion adsorption, organic dye degradation, and oilfield wastewater treatment. By optimizing the structure and function of the materials, the researchers have successfully improved water treatment efficiency and reduced treatment costs, providing strong support for the protection and sustainable use of water resources.

Future research should focus on three major directions. In terms of material performance optimization, it is necessary to break through the bottleneck of anti-pollution and the stability of membrane materials, develop smart response materials with high selectivity and low energy consumption, and explore the scale-up processing technology of MOFs/COFs hybrid systems. At the level of technology integration and innovation, we can deepen the integration of machine learning and experimental data, construct adsorption kinetic prediction models, and expand multi-material synergistic systems (such as magnetic separation and photocatalytic coupling) combined with green synthesis processes to reduce energy consumption and carbon footprint. Engineering applications need to strengthen the validation of complex scenarios: assessing the risk of particle shedding in the long-term operation of photocatalytic membranes, establishing molecular weight efficacy correlation models of scale inhibitors to guide industrial selection, and optimizing the replacement cycle of carriers to enhance system stability.

Furthermore, there is an urgent need to improve sustainability assessment systems, including full life cycle analysis of bio-based materials, environmental impact assessment of low-toxicity modification strategies, and research on the economics of recycling materials. Through interdisciplinary collaboration and data-driven design, functional polymer materials are expected to drive water treatment technologies toward the goals of high efficiency, intelligence, and carbon neutrality, and provide innovative solutions to the global challenge of clean drinking water outlined in the United Nations 2030 Sustainable Development Goals. In short, the application of functional polymer materials in the field of water treatment will continue to progress, contributing more wisdom and strength to address global water challenges.

## Data Availability

Not applicable.
